# The impact of *15-minute fitness circles* implemented in China on the public’s subjective well-being–an empirical analysis based on CGSS2021

**DOI:** 10.3389/fpubh.2025.1563722

**Published:** 2025-04-24

**Authors:** Yifei Shen, Long Niu, Li Miao

**Affiliations:** ^1^Department of Physical Education, Xidian University, Xi’an, China; ^2^Department of Sociology, School of Humanities and Social Sciences, Xi’an Jiaotong University, Xi’an, China; ^3^Center for Physical Education, Xi’an Jiaotong University, Xi’an, China; ^4^Department of Liberal Studies, Zhejiang Tongji Vocational College of Science and Technology, Hangzhou, China

**Keywords:** subjective well-being, *15-min fitness circle*, community fitness environment, sport participation, national fitness

## Abstract

**Background:**

Communities are the main places where the public spend their daily activities, and suitable community environments can help improve their subjective well-being. However, there is still limited evidence on how fitness environments in communities affect the public’s subjective well-being. In recent years, Chinese governments have been created the *15-min fitness circle* in communities to provide the public with convenient fitness facilities and venues. Whether this policy is effective in improving the public’s subjective well-being and the mechanisms that how this policy affects it has not been fully explored. To answer this question, we further focus on the mediating role of sports participation between the *15-min fitness circle* and public subjective well-being.

**Methods:**

This study utilizes data from the 2021 China General Social Survey (CGSS) to integrate the *15-min fitness circle*, public sports participation, and subjective well-being into a unified analytical framework. A three-step regression model was used to analyse their relationship and the mediating effect of sport participation. And SPSS Macro PROCESS was used to test the robustness of their mediating effects.

**Result:**

Our research findings indicate the following: (1) the *15-min fitness circle* has a significant positive predictive effect on the subjective well-being of the Chinese public (*β* = 0.080, *p* < 0.001), (2) The *15-min fitness* circles have a significant positive impact on sports participation (β = 0.234, *p* < 0.001), and (3) sports participation serves as a crucial mediator in the relationship between the *15-min fitness circle* and subjective well-being [Bootstrap 95% CI: 0.008, 0.019].

**Conclusion:**

The study concludes that community fitness facilities are of great relevance in improving the subjective well-being of the public. The results of the study provide empirical support for the rationalization of the *15-min fitness circle* in China, and provide theoretical and practical references for other developing countries in exploring ways to improve the subjective well-being of the public.

## Introduction

Well-being refers to individuals’ overall perception and evaluation of their life state and quality of life (1), and the attainment of subjective well-being is a core pursuit in people’s lives. According to the World Happiness Report (2), China’s happiness index stands at 5.973, ranking 60th among nearly 140 surveyed countries and regions, an improvement of four places compared to the World Happiness Report 2023. However, a survey conducted by the World Health Organization (WHO) indicates that with the rapid economic development of nations, significant changes have occurred in daily life domains such as transportation modes, work patterns, and dietary habits. These shifts have inadvertently contributed to increased sedentary behavior, with up to 70% of individuals reportedly engaging in insufficient physical activity (3). Over time, these trends have not only led to a rise in obesity rates (4) but also exacerbated the prevalence of cardiovascular diseases, diabetes, and other chronic conditions (5, 6), posing severe threats to public health and diminishing overall well-being. Consequently, in the context of fast-paced modern life, the question of how to provide more accessible opportunities for physical exercise, thereby enhancing public health and subjective well-being, has become a critical issue for both the Chinese government and the academic community.

To address the practical challenge of “where to exercise” faced by the public, the Chinese government issued the Opinions on Accelerating the Development of the Sports Industry and Promoting Sports Consumption as early as 2014 (7). This policy emphasized the need for a scientifical plan and strategical distribution of the sports facilities, including the creation of a *15-min fitness circle* within urban communities. The initiative aimed to ensure that within a 15-min walking or cycling radius from their residences, residents could access fitness venues suitable for activities such as walking, square dancing, and ball games. The policy also mandated full coverage of sports facilities in newly built communities. Subsequently, in 2016, the government released the National Fitness Plan (2016–2020) (8), which further advocated for the establishment of a three-tier fitness facility network across counties, towns, and villages, as well as the implementation of the urban *15-min fitness circle*, to facilitate convenient access to physical exercise for residents. Additionally, the Healthy China 2030 Plan and subsequent National Fitness Plans reiterated the importance of promoting the development of sports infrastructure as a key objective (9). In summary, these policy measures reflect the Chinese government’s concerted efforts to address the shortage of recreational and fitness spaces caused by rapid social development, aiming to enhance public access to sports and fitness opportunities.

From the perspective of academic research, the impact of sports facilities in the residential environment on the public’s subjective well-being has received equal attention from scholars in recent years. Existing studies suggest that communities, as spatial carriers of residents’ living and daily activities, are closely linked to public well-being through their nearby facilities. The presence of parks, recreational spaces, and fitness facilities in the vicinity of a community can encourage physical and outdoor activities (10, 11), reduce sedentary behaviors, and address issues such as obesity (12) and chronic diseases (13). These improvements contribute to better physical health (14) and enhance subjective well-being (15). Furthermore, the ecological model theory posits that environmental barriers to physical activity are significant factors influencing sports participation (16). For instance, a study from South Korea found that better sports facilities were associated with higher levels of physical activity among adults (17). Similarly, a cross-sectional cohort study in Finland revealed that when individuals moved to areas with fewer sports facilities (e.g., increased distance or reduced availability), their participation in physical activities declined (18). Moreover, research indicates that people tend to prefer open outdoor spaces for physical activities (19, 20), and individuals who engage in frequent physical activities report greater life satisfaction and happiness compared to those who are inactive (21). It can be seen that having a convenient environment of fitness facilities around the community is very important for the participation rate of physical activity and the subjective well-being of residents (22).

Although previous studies have shown the impact of living environment on public subjective well-being and noticed the importance of having entertainment spaces or fitness facilities near the community, there is still room for further researches. Firstly, from the current literature on the subject, it can be found that the empirical data of the existing studies come from developed countries, however, the relevant studies in China are more limited, especially in the context of the *15-min fitness circle* policy, and there is a lack of empirical studies to explore the effects and mechanisms of fitness facilities on the subjective well-being of the public. Second, it has been 10 years since the Chinese government formally proposed in 2014 to continuously improve fitness facilities for the whole population and create 15-min fitness circles in communities across China, but its impact on the public’s subjective well-being has not been adequately investigated. This study focuses on the impact of fitness facilities on public subjective well-being in Chinese communities in the context of China, and focuses on the following two questions. First, does the existence of a *15-min fitness circle* affect the public’s subjective well-being? Second, what paths does the *15-min fitness circle* take to influence the public’s subjective well-being? By exploring the impact of the implementation of the *15-min fitness circle* policy on the public’s subjective well-being and the pathways through which it affects the public’s subjective well-being, this study not only provides empirical evidence for China’s national fitness policy, but also serves as a reference for the design and implementation of national fitness policies in other countries.

## Literature review and research hypotheses

### The *15-minute fitness circle* and subjective well-being

The study of well-being originated in the United States during the 1950s, with its initial conceptualization emerging in psychology. Over time, the concept has been adopted and explored across various disciplines, including economics, sociology, and sports science, gradually becoming a prominent research focus. Assessments of well-being are multidimensional, encompassing aspects such as subjective well-being and life satisfaction (23, 24). Currently, scholarly attention is predominantly directed toward subjective well-being. Scholars have posited that subjective well-being is often described as an individual’s subjective perception and emotional response resulting from interactions with external environments, influenced by the availability of environmental resources and closely linked to personal behaviors (25). Empirical studies have demonstrated a significant relationship between subjective well-being and residential environments (26, 27). Specifically, residential environments substantially impact the comfort, convenience (28), and promotion of healthy lifestyles (29) in daily life. And convenience, as an important factor in promoting public participation in social activities, has a positive effect on improving the public’s mental health and healthy lifestyle (30). It is mainly reflected in the supporting facilities around the residential area, such as public transportation and barrier-free facilities, and how much convenience they can provide for the public’s daily life (31). Additionally, a study by Ma et al. (32) focusing on Sydney, Australia, found that the convenience and accessibility of public facilities enhance subjective well-being. Rowe et al. (33) further refined this focus to examine public sports facilities, concluding that the availability of public sports infrastructure near residential areas positively impacts subjective well-being. Although existing research highlights the influence of fitness facilities on subjective well-being, most studies have been conducted in developed countries, leaving research from developing countries relatively scarce. Scholars in sociology have emphasized the need for future research to examine the impact of urban public sports facilities on subjective well-being, particularly in the context of developing nations (34). Given the policy-driven effort in China to establish community fitness environments, including the *15-min fitness circle*, this study explores its impact on the subjective well-being of Chinese residents. The investigation aims to fill a critical research gap and provide practical implications. Hypothesis 1 is formulated in this study:

*H1*: The *15-min fitness circle* has a significant positive impact on the subjective well- being of the Chinese public.

### The impact of sports participation on subjective well-being

To date, substantial research has been conducted on the relationship between sports participation and subjective well-being. For instance, as early as 1974, Snyder and Spritzer (35) found that sports participation not only fosters social interactions but also generates a sense of pleasure during physical activity, thereby enhancing individual well-being. This seminal finding laid the foundation for subsequent research in this field, sparking widespread scholarly interest. Pawlowski et al. (36) conducted a cross-national study involving 19 European countries, demonstrating that individuals across different nations and regions experience significant improvements in subjective well-being through participation in sports activities. Similarly, a study in Germany corroborated this conclusion, reporting that sports participation has a markedly positive effect on public well-being (37). Further analysis by Downward and Rasciute (38) examined the effects of participation in 67 different types of sports activities on subjective well-being, controlling for individuals’ socio-demographic characteristics. The results revealed that individuals who engaged in sports activities were happier and reported higher levels of subjective well-being (38). The underlying mechanisms for this relationship include improvements in physical health, the alleviation of stress and external pressures, the reduction of depressive symptoms, and the enhancement of physical fitness through sports participation (39, 40). Moreover, the process of engaging in sports fosters social interaction and communication, enabling individuals to build social capital such as social trust and support (41). These factors collectively contribute to higher levels of well-being ((42)). Based on these findings, we propose Hypothesis 2:

*H2*: Sports participation has a significant positive impact on the subjective well-being of the public.

### The *15-minute fitness circle*, sports participation, and subjective well-being

Sports fitness facilities serve as the foundational infrastructure enabling the public to engage in physical exercise. Over the past decades, as numerous countries have invested heavily in sports facilities to promote physical activity, scholars have increasingly turned their attention to the relationships among fitness facilities, sports participation, and subjective well-being. On one hand, in studies examining sports facilities and sports participation, the ecological model theory posits that sports participation typically occurs in specific venues for physical activities, and environmental barriers may significantly influence participation rates (16). Empirical research supports this notion, demonstrating that the availability of sports facilities near one’s residence—a key environmental factor—has a substantial impact on individual engagement in physical exercise (43). For instance, Heinrich et al. (44) found that the availability of sports facilities encourages higher participation in sports activities. Similarly, Marijke et al. (45) highlighted that recreational facilities not only increase physical activity levels among adults but also improve their overall health outcomes. In Brazil, a nationwide telephone survey (VIGITEL) revealed that adults are 67% more likely to engage in physical activities during their leisure time if adequate facilities are available near their homes (46). Additionally, a study conducted in Denmark using GPS and accelerometers echoed these findings, showing that the presence of sports facilities within 800 meters of one’s residence is associated with longer durations of moderate-to-vigorous physical activity (47). On the other hand, research exploring the relationship among fitness facilities, sports participation, and subjective well-being remains relatively sparse. While some studies have indicated that individuals living closer to fitness facilities tend to participate more in sports activities and report higher levels of happiness (48), these studies have not conducted an in-depth analysis of the mediating mechanisms. According to the three-dimensional framework of ‘environment-process-person’ proposed by Scannell et al. (49), which states that the ‘process’ consists of three dimensions: cognitive, behavioral and affective, the dimension of ‘process’ was chosen as the intermediate variable in this study (49), we propose Hypotheses 3 and 4:

*H3*: The *15-min fitness circle* has a significant positive impact on public sports participation.

*H4*: Sports participation plays a significant mediating role in the relationship between the *15- min fitness circle* and subjective well-being.

## Methods

### Data sources

This study utilizes data from the 2021 Chinese General Social Survey (CGSS) to empirically test the aforementioned propositions. The Chinese General Social Survey, designed, implemented, and disseminated by the National Survey Research Center (NSRC) at Renmin University of China, is the first national, comprehensive, and continuous large-scale social survey project in China. It is widely recognized and extensively applied in the field of social science research. The 2021 CGSS collected a total of 8,148 valid samples, covering 19 provinces in China, with the target population being adults aged 18 and above. The CGSS 2021 survey covers variables related to residents’ subjective well-being, the 15-min fitness circle, and sports participation, and is one of the most authoritative and representative national survey data available in China. The key variable 15-min fitness circle used in this study is located in module G of the questionnaire, which was completed by a total of 2,717 respondents. After removing missing values and invalid samples, 2,706 valid samples were finally obtained for data analysis. Among them, males account for 45.20% (1,223 individuals), while females make up 54.80% (1,483 individuals), ensuring national representativeness.

### Measures

#### Dependent variable

The dependent variable in this study is subjective well-being. Well-being can be measured using either subjective or objective criteria (50), with this study focusing on subjective well-being. Scholars often employ univariate measures and self-report methods when assessing happiness indices (51). Research has shown that self-reported subjective well-being data exhibit high levels of consistency, reliability, and stability (52). In the CGSS 2021 survey questionnaire, respondents were asked: “Overall, do you feel happy with your life?” There were seven response options: 1 = “Very unhappy,” 2 = “Relatively unhappy,” 3 = “Neither happy nor unhappy,” 4 = “Relatively happy,” 5 = “Very happy,” 98 = “Do not know,” and 99 = “Refused to answer.” Responses coded as 98 and 99 were treated as missing values and subsequently excluded from the analysis. Higher scores indicate a higher level of subjective well-being among respondents.

#### Independent variable

The independent variable in this study is the public’s evaluation of the *15-min fitness circle*. In the CGSS 2021 survey, there is a question that asks: “Within a 1-kilometer radius (approximately a 15-min walk) from my residence, there are suitable places for physical exercise, such as jogging, walking, etc.” Respondents were given five options: 1 = “Strongly agree,” 2 = “Agree,” 3 = “Neither agree nor disagree,” 4 = “Disagree,” and 5 = “Strongly disagree,” For the analysis, the responses were reverse-coded, where 1 = “Strongly disagree,” 2 = “Disagree,” 3 = “Neither agree nor disagree,” 4 = “Agree,” and 5 = “Strongly agree.”

#### Mediating variables

He mediating variable in this study is sports participation. For the purposes of this study, public sports participation is limited to the dimension of “direct participation,” referring spe-cifically to active engagement in sports activities. In the survey, respondents were asked: “Over the past year, how frequently have you engaged in sports activities during your leisure time?” The response options were as follows: 1 = “Daily,” 2 = “Several times a week,” 3 = “Several times a month,” 4 = “Several times a year or less,” 5 = “Never” and 98 = “Do not know.” To facilitate analysis, the responses were reverse-coded as follows: 1 = “Never,” 2 = “Several times a year or less,” 3 = “Several times a month,” 4 = “Several times a week,” 5 = “Daily” and eliminated 98 = “do not know.” Higher values indicate greater frequency of sports participation.

#### Control variables

We controlled for demographic variables, including respondents’ gender, age, education level, income, household registration location, physical health, and marital status. Among these, gender (“male” = 0, “female” = 1), education level (“low education level” = 0, “high education level” = 1), household registration location (“rural” = 0, “urban” = 1), and marital status (“unmarried” = 0, “married” = 1) were coded as dummy variables. For income, we used respondents’ annual income and applied mean imputation using monthly income for missing values. Additionally, annual income was log-transformed. Physical health status was treated as a continuous variable (“very unhealthy” = 1, “relatively unhealthy” = 2, “moderate” = 3, “relatively healthy” = 4, “very healthy” = 5).

### Statistical analysis

The analysis was conducted with Stata 17.0 for descriptive statistical analysis and correlation analysis. Subsequently, a three-step regression was conducted to analyze the relationships between the *15-min fitness circle*, sports participation, and subjective well-being, while controlling for gender, age, education level, income, household registration, physical health and marital status. The mediating role of sports participation in the relationship between the *15-min fitness circle* and subjective well-being was also tested. To ensure the robustness of the mediation analysis, SPSS 27.0 was employed with Model 4 of the PROCESS macro developed by Hayes (63). Using the bootstrap method for repeated sampling of the dataset, we estimated the direct and indirect effects and constructed confidence intervals for the mediation effects. A mediation effect was considered statistically significant if the confidence interval did not include zero.

## Results

### Descriptive statistics

Table 1 presents the descriptive statistics of the key variables. The respondents’ ages ranged from 18 to 99 years, with a mean age of 52 years. Female respondents accounted for 54.8% of the sample, while male respondents made up 45.2%. The overall education level was relatively low, with 61.83% of respondents having completed middle school or less, and only 38.17% having attained a high school education or above. After applying a natural logarithmic transformation to income, the values ranged from 0 to 13.71. The majority of respondents resided in rural areas compared to urban areas. The overall health status is relatively good, and the majority of residents are married. The average score for sports participation was 2.829 (SD = 1.620). Notably, the subjective well-being of the Chinese public was relatively high, with an average score of 3.980 (SD = 0.823). Similarly, the coverage of the *15-min fitness circle* was substantial, with an average score of 3.815 (SD = 1.013).

**Table 1:** Basic variable description statistics table.

Variable	Obs	Mean	Std	Min	Max
Subjective well-being	2,706	3.980	0.823	1	5
*15-minute fitness circle*	2,706	3.815	1.013	1	5
Sports participation	2,706	2.829	1.620	1	5
Gender	2,706	0.548	0.498	0	1
Age	2,706	52.035	17.643	18	99
Education	2,706	0.382	0.486	0	1
Income	2,706	8.297	4.070	0	13.71
Urban	2,706	0.397	0.489	0	1
Health	2,706	3.468	1.085	1	5
Marriage	2,706	0.851	0.356	0	1

### Correlation analysis

As shown in Table 2, there is a positive correlation between the public’s subjective well-being and the *15-min fitness circle*, sports participation, age, education level, income, household registration, and physical health, indicating that the *15-min fitness circle* and sports participation contribute to improving the public’s subjective well-being. The 15-min fitness circle is positively correlated with sports participation, education level, and physical health. Additionally, sports participation is positively correlated with education level, income, household registration, and physical health, while it is negatively correlated with gender, age, and marital status.

**Table 2:** Pearson’s correlations among relevant study variables.

Variables	Subjective well-being	*15-minute fitness circle*	Sports participation	Gender	Age	Education	Income	Urban	Health	Marriage
Subjective Well-being	1									
*15-minute fitness circle*	0.120***	1								
Sports participation	0.160***	0.171***	1							
Gender	−0.024	−0.023	−0.070***	1						
Age	0.044*	−0.025	−0.098***	−0.034	1					
Education	0.071***	0.073***	0.272***	−0.087***	−0.463***	1				
Income	0.087***	0.034	0.102***	−0.221***	−0.018	0.201***	1			
Urban	0.085***	0.034	0.229***	−0.038*	−0.010	0.386***	0.248***	1		
Health	0.221***	0.069***	0.142***	−0.043*	−0.376***	0.236***	0.093***	0.061**	1	
Marriage	0.034	0.001	−0.106***	0.070***	0.503***	−0.347***	0.044*	−0.048*	−0.194***	1

*** *p* < 0.001, ** *p* < 0.01, * *p* < 0.05.

### The effect of a 15-min fitness circle on subjective well-being

Table 3 presents the main model, which employs three-step regression to examine the mediating effect of sports participation in the relationship between the *15-min fitness circle* and subjective well-being. Model 1 represents the first step of the mediation analysis, assessing the direct impact of the *15-min fitness circle* on subjective well-being. The results indicate that the *15-min fitness circle* has a significant positive effect on subjective well-being (*β* = 0.080, *p* < 0.001). This finding suggests a strong association between the two variables; in other words, the presence of fitness facilities within close proximity to a community enhances the subjective well-being of its residents. Thus, Hypothesis 1 is supported. In addition, several control variables were found to significantly influence subjective well-being, including age (β = 0.008, *p* < 0.001), education level (*β* = 0.113, *p* < 0.01), income (β = 0.009, *p* < 0.05), and physical health (β = 0.197, *p* < 0.001). These findings align with previous research. For instance, Kang et al. (53) found that older adults report higher levels of subjective well-being compared to middle-aged and younger individuals, and individuals with higher income levels also exhibit greater subjective well-being (53).

**Table 3:** Results of mediation analysis of subjective well-being.

	Model 1 subjective well-being	Model 2 sports participation	Model 3 subjective well-being
*15-minute fitness circle*	0.080*** (0.015)	0.234*** (0.029)	0.067*** (0.015)
Sports participation			0.055*** (0.010)
Gender (male)			
Female	0.015 (0.032)	−0.113 (0.061)	0.022 (0.031)
Age	0.008*** (0.001)	0.004 (0.002)	0.007*** (0.001)
Education (low level of education)			
High level of education	0.113** (0.040)	0.629*** (0.077)	0.078 (0.040)
Income	0.009** (0.004)	0.004 (0.008)	0.008* (0.004)
Domicile (rural)			
Urban	0.056 (0.035)	0.465*** (0.067)	0.030 (0.035)
Health	0.197*** (0.015)	0.128*** (0.029)	0.190*** (0.015)
Marriage (unmarried)			
Married	0.054 (0.050)	−0.169 (0.097)	0.064 (0.050)
_cons	2.403 (0.112)***	1.023 (0.215)***	2.346 (0.111)***
*N*	2,706	2,706	2,706
pseudo *R*^2^	0.086	0.121	0.096

Standard errors in parentheses * *p* < 0.05, ** *p* < 0.01, *** *p* < 0.001.

Model 2 represents the second step of the mediation analysis, examining the effect of the *15-min fitness circle* on sports participation. The results demonstrate that the *15-min fitness circle* significantly promotes sports participation (*β* = 0.234, *p* < 0.001), providing support for Hypothesis 3. Furthermore, the analysis results indicate that residents with higher education levels, urban household registration, and better physical health exhibit higher levels of sports participation. However, the effects of age, gender, income, and marital status on sports participation were not statistically significant.

**Figure 1:**
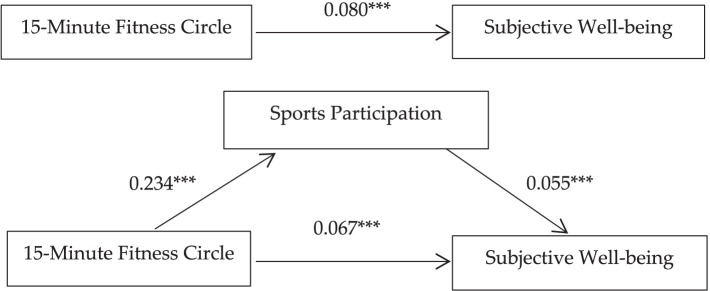
Mediating effects pathway diagram.

Model 3 represents the third step of the mediation analysis, incorporating both the *15-min fitness circle* and sports participation into the model. The results indicate that sports participation has a significant positive effect on subjective well-being (*β* = 0.055, *p* < 0.001), while the 15-min fitness circle continues to exert a significant positive impact on subjective well-being (β = 0.067, *p* < 0.001). Notably, the coefficient of the *15-min fitness circle* in the third step is smaller than that in the first step, and the coefficient of the mediating variable (sports participation) remains significant. This finding suggests that sports participation partially mediates the relationship between the *15-min fitness circle* and subjective well-being. Thus, both Hypothesis 2 and Hypothesis 4 are supported. In order to visualize the mediating effects of sports participation more visually, we created a pathway diagram (Figure 1).

### Robustness check

To further validate the reliability of the empirical analysis, robustness testing was conducted using SPSS 27.0 with Model 4 of the PROCESS macro developed by Hayes (2017). As shown in Table 4, the total effect analysis reveals that the *15-min fitness circle* has a significant positive impact on subjective well-being [Bootstrap 95% CI: 0.051, 0.109]. The direct effect results indicate a positive relationship between the *15-min fitness circle* and subjective well-being [Bootstrap 95% CI: 0.037, 0.097]. Furthermore, the indirect effect analysis shows that sports participation significantly enhances subjective well-being [Bootstrap 95% CI: 0.008, 0.019]. These results are consistent with the findings from the stepwise regression analysis, supporting the robustness of our study’s conclusions.

**Table 4:** Results of the mediating effect of sports participation.

Paths	Standardized coef.	Bootstrap 95%CI	
		Lower	Upper
Total effect			
15-Minute Fitness circle → subjective well-being	0.080	0.051	0.109
Direct effects			
15-Minute fitness circle → subjective well-being	0.067	0.037	0.097
Indirect effects			
15-Minute fitness circle →sports participation →Subjective well-being	0.013	0.008	0.019

## Discussion

Against the backdrop of the Chinese government’s efforts to build community fitness facilities, this study analyzes the impact of China’s *15-min fitness circle* on the public’s subjective well-being based on data from the China General Social Survey (CGSS2021). And it further validates the potential mechanisms behind this process. The findings reveal that the *15-min fitness circle* enhances public subjective well-being by promoting sports participation. Several important conclusions can be drawn from this study.

First, we found a positive correlation between the *15-min fitness circle* and public subjective well-being. This result aligns with previous studies on the impact of fitness facilities (54). Specifically, as China transitions from rapid economic growth to high-quality development, this shift is reflected in the improvement of living environments and the rising expectations and satisfaction of residents for a better quality of life. In this context, convenient fitness facilities within communities serve as essential resources for encouraging public participation in physical exercise and enhancing physical health. These facilities not only meet the practical needs of individuals seeking a healthy lifestyle but also contribute significantly to promoting subjective well-being. Furthermore, existing studies suggest that inequalities in the availability of fitness facilities can limit access for socioeconomically disadvantaged groups, thereby increasing the risk of obesity (55). However, reducing social exclusion has been shown to enhance individual well-being within communities (56). A similar phenomenon exists in China, where the “urban–rural dual structure” has long created disparities in socioeconomic status and access to health resources. By equitably allocating community fitness facilities through initiatives like the *15-min fitness circle*, these gaps in health resources between urban and rural areas, as well as across socioeconomic groups, can be narrowed. This approach promotes health equity, strengthens residents’ sense of belonging and identification with their communities, and ultimately enhances public subjective well-being.

Second, sports participation is positively correlated with public subjective well-being, a finding consistent with prior research. For instance, Piqueras et al. (57) reported that individuals who regularly engage in sports activities exhibit subjective well-being levels that are 1.3 times higher than those of their peers who do not participate in such activities. This relationship is not surprising given the demands of modern life, where increasingly heavy workloads and associated pressures contribute to rising levels of stress and anxiety, which erode both physical and mental health—key determinants of subjective well-being. As a source of both health and happiness, sports activities offer multiple benefits. They not only improve physical health (58) but also help individuals manage negative emotional states, such as irritability and depression, caused by social stressors. By alleviating these adverse effects, sports participation fosters a positive outlook on life, thereby exerting a significant positive impact on subjective well-being.

Finally, this study identifies a significant mediating effect of sports participation in the relationship between the *15-min fitness circle* and subjective well-being. This finding not only underscores the critical role of fitness facilities in promoting sports participation and subjective well-being but also provides valuable insights for the planning and policy optimization of China’s *15-min fitness circle* initiative. While some studies have indicated that most individuals engage in sports activities beyond an 800-meter radius from their homes (59), the majority of evidence supports the notion that recreational facilities in close proximity to residential areas increase public participation in sports (48, 64). Our study further corroborates this conclusion, demonstrating that individuals living closer to fitness facilities are more likely to engage in sports and report higher levels of subjective well-being. This finding can be attributed, on one hand, to the diversity and adaptability of fitness facilities within China’s 15-min fitness circles. Chinese scholars Zhang et al. (60) and Wang et al. (20) have highlighted that the variety of fitness facilities significantly impacts subjective well-being, as the diversity of these facilities determines the range of fitness functions available within a community. This multifunctionality meets diverse needs for physical exercise and social interaction, thereby enhancing subjective well-being (60). Specifically, the *15-min fitness circle* implemented by the Chinese government encompasses a wide range of facilities, including outdoor fitness equipment, basketball courts, soccer fields, table tennis tables, fitness trails, and bike lanes. These diverse facilities cater to the fitness needs of individuals across various age groups, income levels, and occupational categories, enabling people to engage in activities that suit their specific preferences and requirements, thereby improving their subjective well-being. On the other hand, the construction of the *15-min fitness circle* addresses the varying time constraints of different demographic groups, providing an essential pathway for equitable allocation of public time resources. In the context of the fast-paced lifestyle of modern society, the accessibility and proximity of such facilities reduce both the time and economic costs associated with participating in sports activities. This, in turn, fosters greater engagement in physical exercise, allowing more individuals to integrate a healthy lifestyle into their daily routines. Furthermore, the *15-min fitness circle* contributes to cultivating a vibrant and positive sports culture, acting as a social anchor within communities (61). This initiative not only promotes stronger neighborhood relationships and the accumulation of social capital (62) but also enhances residents’ sense of belonging to their communities, thereby exerting a positive influence on their subjective well-being.

The findings of this study have important implications for public policymakers and urban planning practices. The significant impact of the *15-min fitness circle* on public subjective well-being highlights the importance of improving the accessibility of community sports facilities. By scientifically planning facility distribution and enhancing their utilization, policymakers can effectively promote residents’ participation in physical activities, thereby improving overall well-being. Moreover, the mediating role of sports participation suggests that policy design should not only focus on infrastructure development but also actively promote community fitness programs and activities to encourage residents to engage in regular exercise. Despite its contributions, this study has certain limitations that should be addressed in future research. First, due to data constraints, the measurement of the *15-min fitness circle* in this study relies solely on respondents’ self-assessments, lack of objective measurements in the field. Second, this study is based only on data from the 2021 wave of the China General Social Survey (CGSS) and does not incorporate data from other years, making it difficult to capture the dynamic impact of the *15-min fitness circle* on public subjective well-being over time. Third, while the path analysis provides an initial interpretation of the relationships between variables, causal inferences should be made with caution. Future research could adopt longitudinal or experimental designs and complement them with qualitative methods, such as interviews, to further explore the underlying mechanisms of the findings and enhance the explanatory power and depth of the study.

## Conclusion

This study, based on data from the China General Social Survey (CGSS) 2021, integrates the *15-min fitness circle*, sports participation, and subjective well-being within a single framework to systematically explore the impact of community sports facilities on public subjective well-being and its underlying mechanisms. The findings reveal three key insights: first, the presence of a *15-min fitness circle* significantly enhances public subjective well-being; second, sports participation has a significant positive impact on subjective well-being; third, sports participation plays a significant mediating role between the *15-min fitness circle* and subjective well-being. These findings provide empirical support for China’s community-based 15-min fitness circle policy, indicating that convenient sports facilities are not only an important material foundation for promoting public sports participation but also a crucial safeguard for improving quality of life and enhancing subjective well-being. Our results offer valuable theoretical and practical insights for other developing countries looking to improve public subjective well-being through community sports facilities. Future research could further explore the long-term effects of community fitness facilities on different age groups and examine the moderating role of cultural or socio-economic factors.

## Data Availability

The original contributions presented in the study are included in the article/supplementary material, further inquiries can be directed to the corresponding author.
